# Comparative sub-chronic toxicity studies in rats of two indistinguishable herbal plants, *Cynanchum wilfordii* (Maxim.) Hemsley and *Cynanchum auriculatum* Royle ex Wight

**DOI:** 10.1007/s10068-022-01072-5

**Published:** 2022-04-19

**Authors:** Changwoo Yu, So-Hye Hong, Jin Hee Lee, Ki Kyung Jung, Jae-Ho Oh, Jayoung Jeong, HoonJeong Kwon, Jong-Koo Kang, Jun-Young Yang

**Affiliations:** 1 Toxicology Research Division, Ministry of Food and Drug Safety, National Institute of Food and Drug Safety Evaluation, 187, Osongsaengmyeong 2-ro, Osong-eup, Heungdeok-gu, Cheongju-si, Chungcheongbuk-do 28159 Republic of Korea; 2 Department of Food and Nutrition, Seoul National University, 1, Gwanak-ro, Gwanak-gu, Seoul, 08826 Republic of Korea; 3 Research Institute of Human Ecology, Seoul National University, 1, Gwanak-ro, Gwanak-gu, Seoul, 08826 Republic of Korea; 4 Biotoxtech Co., Ltd., 53, Yeongudanji-ro, Ochang-eup, Cheongwon-gu, Cheongju-si, Chungchcengbuk-do 28115 Republic of Korea; 5 Department of Laboratory Animal Medicine, College of Veterinary Medicine, Chungbuk National University, 1, Chungdae-ro, Seowon-gu, Cheongju-si, Chungchengbuk-do 28644 Republic of Korea

**Keywords:** *Cynanchum wilfordii* (Maxim.) Hemsley, Baeksuo, *Cynanchum auriculatum* Royle ex wight, Iyeopupiso, Sub-chronic toxicity

## Abstract

Sub-chronic toxicity studies using rats have been conducted for *Cynanchum wilfordii* (Maxim.) Hemsley (CW) and *Cynanchum auriculatum* Royle ex Wight (CA). CW water extract didn’t show any adverse effects whereas administering CW powder decreased body weights in complication with decreased food consumptions. In the case of CA water extract, triglyceride and absolute/relative liver weights were elevated and vacuolation was observed in liver. Treated CA powder in male rats increased alanine aminotransferase and aspartate aminotransferase and induced single cell necrosis and multinucleated hepatocyte in liver. As for female rats, increased absolute/relative weights and hypertrophy/vacuolation in adrenal glands and vacuolation in ovaries were observed when administered CA powder. In conclusion, no observed adverse effect level (NOAEL) of CW water extract was over 5000 mg/kg/day, while NOAEL of CW powder was 700 mg/kg/day for female and 150 mg/kg/day for male. In case of CA, NOAEL of water extract was 1500 mg/kg/day for male and 2000 mg/kg/day for female, while NOAEL of powder was 150 mg/kg/day for both gender. To the best of our knowledge, this is the first sub-chronic toxicity study on the adverse effects, target organs and its dose levels of *C. wilfordii* (Maxim.) Hemsley and *C. auriculatum* Royle ex Wight following GLP protocols.

## Introduction

Root parts of *Cynanchum wilfordii* (Maxim.) Hemsley (CW) cv. ‘Baeksuo’, have long been used as traditional herbal medicines in East Asia including Korea. Based on the Korean traditional medical literatures, although its name was first appeared in 1959, it was thought that CW had been used in Korea since at least 1600 s (Choi et al., 2003). From the past, CW was used for its remedial effects such as invigorating body, nourishing blood, improving blood circulation, strengthening muscle and bone, preventing from aging and curing neurasthenia (MFDS, 2014a). Also, recent scientific studies revealed health benefits of CW. Several researchers showed that CW and its components like cynandione A, wilfoside K1N or steroidal compounds have effects of anti-inflammation (Cho et al., 2017; Kim et al., 2015; Koo et al., 2015; Yang et al., 2014), anti-cancer (Lee et al., 2017), liver protection (Jang et al., 2016), scavenging free radicals (Jang et al., 2017), preventing liver from accumulation of fats (Kim et al., 2020), suppressing LDL modification (Kim et al., 2019), relieving lipid related symptoms or diseases (Lee et al., 2013; Youn et al., 2019) and so on. Moreover, considering its medicinal effects of relieving menopausal symptoms like depression, hypertension, forgetfulness and so on (Lee et al., 2016; 2020; Kim et al., 2017), CW extract complex was approved as functional food (food supplement) in Korea.

However, a food safety incident was reported in May, 2015. Following the food safety inspection, it was revealed that most of the CW ingredient was composed of *Cynanchum auriculatum* Royle ex Wight (CA) cv. Iyeopupiso, in place of CW. CA is not approved as herbal medicine in Korea. However, both CW and CA belong to the Asclepiadaceae family and have so similar appearances that even experts can barely distinguish between the two plants with the naked eye. Moreover, in China, both CW and CA was called as the same name and CA was registered as edible food source (Chen et al., 2019). In terms of economic efficiency, farmers preferred CA because it grows faster and bigger than CW (Kim et al., 2005).

The problem is that only few toxicity studies for CW and CA have been conducted and published. In addition, reliable toxicity tests following GLP protocol on CW or CA were not available. Because of these reasons, both of their toxicities were rarely known. To address the public safety issues, toxicity testing in rats has been conducted by Ministry of Food and Drug Safety in Korea in cooperation with Biotoxtech Co. following the safety incident in 2015 (MFDS, 2017). The aim of this report is to summarize the results of 90-day sub-chronic toxicity tests on *C. wilfordii* (Maxim.) Hemsley and *C. auriculatum* Royle ex Wight.

## Materials and methods

### Sample preparation

The root parts of *C. wilfordii* (Maxim.) Hemsley (CW) and *C. auriculatum* Royle ex Wight (CA) were used for toxicity tests. CW and CA were cultivated in 2015 from different regions in Korea, Yeongju-si (Gyeongsangbuk-do, Republic of Korea) and Jecheon-si (Chungcheongbuk-do, Republic of Korea) respectively, to avoid cross contamination. Before preparing samples, every raw material used in the species was validated using evaluation of the appearance by expert group and polymerase chain reaction (PCR)(Biometra, Jena, Germany) in accordance with Korean herbal pharmacopoeia (MFDS, 2014c). Unmatched raw materials were excluded from the test.

**Fig. 1:**
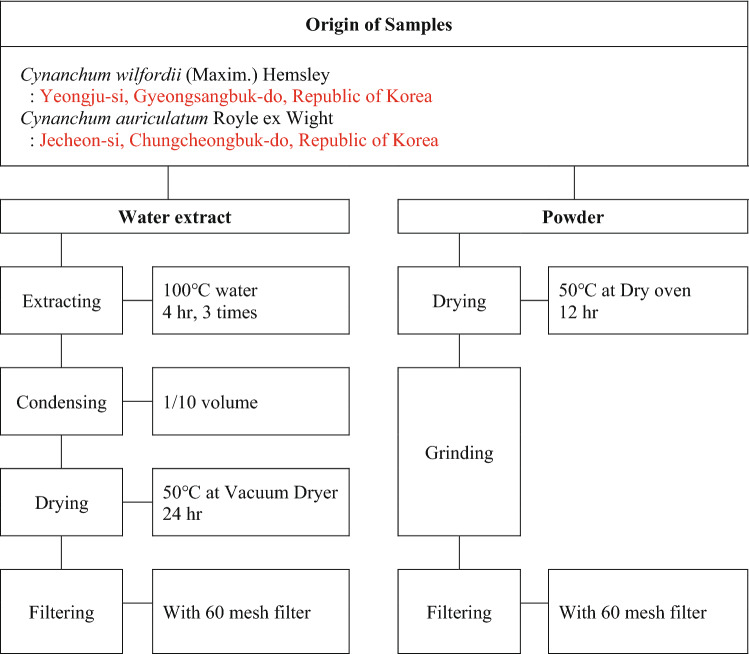
Sample preparation workflow of *Cynanchum wilfordii* (Maxim.) Hemsley and *Cynanchum auriculatum* Royle ex Wight

Samples were supplied by ‘National Development Institute of Korean Medicine’. Because CW and CA were traditionally consumed in extracted or grinded forms, each raw material was prepared in two sample types, water extract and powder. To acquire water extract sample, raw material was extracted thrice at 100 °C, condensed, dried 24 h at 50 °C in vacuum dryer and filtered. In case of powder sample, raw material was dried in oven at 50 °C for 12 h, grinded and filtered (Fig. 1). Samples were dissolved or suspended in distilled water and refrigerated (not frozen) before administered into the test animals.

### Ethics statement

Each studies were approved by institutional animal care and use committee (IACUC) of Biotoxtech Co. (accredited by AAALAC international in 2008). IACUC numbers for CW water extract and powder sample, and CA water extract and powder sample were 160406, 160852, 160318 and 160854 respectively.

### Study protocol

Toxicity tests were conducted by Biotoxtech Co., in compliance with good laboratory practice (MFDS, 2014b) and standard for toxicity study of pharmaceuticals (MFDS, 2015). Ten specific pathogen-free Sprague–Dawley rats (aged 6 weeks of both sex) were used for each test groups. All samples were administered at 10 mL/kg once daily via oral gavage. Administered dose levels were selected based on the toxicity data acquired from the previous acute and sub-acute toxicity studies that had been conducted before the sub-chronic toxicity test. CW water extract and powder were administered at doses of 500, 1000, 2000 and 50, 150, 500, 700 mg/kg/day respectively. Test groups for CA were given 500, 1000, 2000 of water extract samples and 50, 150, 500 mg/kg/day for powder samples respectively.

Clinical signs were examined every day. Body weight, change of body weight and food consumption were measured weekly. At the end of the test, ophthalmological examination was performed, and then blood and urine samples were collected for hematology, clinical chemistry analysis and urinalysis. All test animals were sacrificed and necropsy was performed to examine necropsy findings, measure organ weights and perform histopathological examination. Organs were weighed and fixed in 10% neutral buffered formalin (except testes fixed in Davison fixative) and histopathological examination were performed.

### Statistical analysis

For the numerical values, appropriate statistical analysis methods were applied using statistical analysis program, SAS (ver.9.3, SAS Institute Inc., USA). Following this, Bartlett test was performed to confirm homogeneity of variances. If the distribution is normal, one-way analysis of variance (ANOVA) and Dunnett’s t-test is performed to figure out the statistical differences. If not, Kruskal–Wallis test, Steel test and Mann–Whitney U-test are used.

## Results and discussion

### *Cynanchum wilfordii* (Maxim.) Hemsley

When administering *C. wilfordii* (Maxim.) Hemsley water extract (CWE) into rats for 90 days, no animal death was observed. In addition, no significant differences or adverse effects were observed in histopathological and necropsy findings, clinical chemistry, hematology and urinalysis data, ophthalmological examination, organ and body weights, clinical signs and food consumptions.

**Fig. 2:**
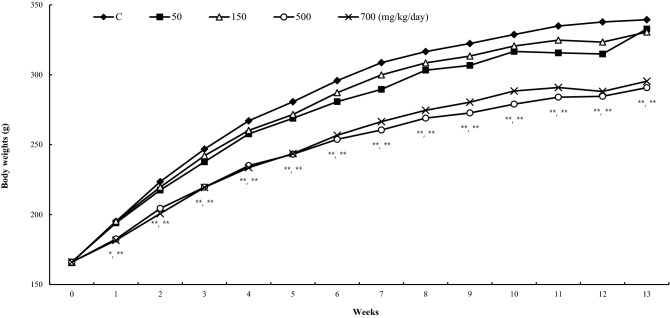
Body weights for female rats in 90-day gavage study of *Cynanchum wilfordii* (Maxim.) Hemsley powder. *,**Significantly different 500 mg/kg/day (left) and 700 mg/kg/day (right) treatment group from control group by Dunnett’s t-test (*p* < 0.05, *p* < 0.01)

Meaningful decreases in body weights were observed when administering *C. wilfordii* (Maxim.) Hemsley powder (CWP) in female rats. Compared to control group (339.4 ± 31.7 g), body weights were decreased by 14.3% (290.8 ± 23.7 g, *p* < 0.01) and 13.0% (295.4 ± 31.0 g, *p* < 0.01) in 500 and 700 mg/kg/day respectively at 13 weeks (Fig. 2). This correlated with declined food consumption in female rats treated with 500 and 700 mg/kg/day in 1–9, 12 weeks and 1–5 weeks respectively (Table 1).

**Table 1:** Body weight changes for rats in 90-day gavage study of *Cynanchum wilfordii* (Maxim.) Hemsley powder

Week	Control	50 mg/kg/day	150 mg/kg/day	500 mg/kg/day	700 mg/kg/day
n		10	10	10	10
Female					
0	21.3 ± 2.0	21.1 ± 1.8	21.3 ± 3.0	20.7 ± 2.2	21.3 ± 2.0
1	22.3 ± 1.0	21.7 ± 2.1	22.5 ± 1.6	18.8 ± 1.7**	18.0 ± 1.8**
2	24.6 ± 2.5	23.6 ± 2.0	23.9 ± 1.9	20.8 ± 1.9**	20.3 ± 1.9**
3	25.3 ± 2.4	24.3 ± 2.3	24.8 ± 2.0	21.5 ± 2.3**	21.3 ± 2.3**
4	26.0 ± 3.1	24.7 ± 2.4	25.4 ± 1.9	21.9 ± 2.1**	22.2 ± 1.7**
5	25.9 ± 2.8	24.8 ± 2.5	24.8 ± 2.1	21.5 ± 2.1**	22.6 ± 2.5*
6	25.7 ± 2.4	24.7 ± 2.8	24.6 ± 2.4	21.7 ± 2.3**	23.0 ± 3.1
7	25.0 ± 2.2	23.8 ± 2.3	24.8 ± 2.8	21.0 ± 1.9**	22.6 ± 2.1
8	24.9 ± 2.3	25.0 ± 3.0	24.6 ± 2.1	21.8 ± 1.7*	23.7 ± 2.4
9	23.3 ± 2.0	23.4 ± 2.5	22.7 ± 2.2	20.4 ± 2.1*	22.8 ± 2.6
10	23.1 ± 1.9	23.6 ± 2.9	23.2 ± 2.4	21.2 ± 1.9	23.0 ± 2.7
11	23.9 ± 2.2	22.8 ± 5.8	23.4 ± 2.0	22.1 ± 2.3	24.6 ± 1.7
12	22.9 ± 1.9	21.2 ± 7.3	21.6 ± 1.9	20.1 ± 1.4^#^	20.9 ± 2.6
13	21.2 ± 1.7	22.7 ± 3.1	21.4 ± 1.5	20.2 ± 1.6	21.8 ± 2.8

*,**Significantly different from control group by Dunnett’s t-test (*p* < 0.05, *p* < 0.01)

^#^Significantly different from control group by Steel test (*p* < 0.05)

Although any adverse effects were not observed after administering CWE, more than 10% of body weight decrease was observed when treating CWP. WHO mentioned that more than 10% of body weight changes could be considered as adverse effect (WHO, 2015). Consequently, it was concluded no-observed adverse effect level (NOAEL) of CWE was 2000 mg/kg/day and NOAEL of CWP was 700 mg/kg/day for males and 150 mg/kg/day for females based on decreased body weights.

Our results share similarities with previous findings that the rats treated with 1% CW of total diets showed decreased body weights at 88.5% (Lee et al., 2013). Liu et al. (2013) also reported one of CW components, wilfoside K1N, even though it was extracted from the CA, could suppress the appetite and decline the body weights in rat. Interestingly, an increase in relative liver weight was reported in some published studies, which was not considered as an adverse effect in this study. According to the EFSA panel on dietetic products, nutrition and allergies (2016), a significant increase in relative liver weights was reported when treating 300 mg/kg/day of micronized powder to female rats. Lee et al. (2013) also showed that treating ethanol extract of CW could increase relative liver weights.

### *Cynanchum auriculatum* Royle ex Wight

**Fig. 3:**
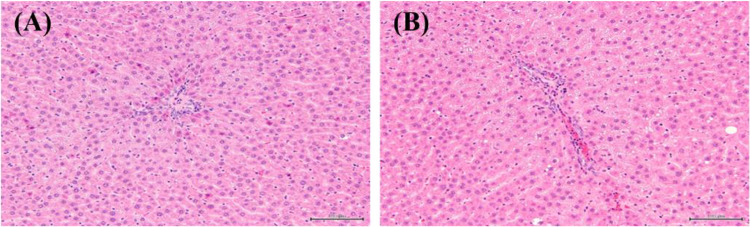
Histopathological examinations treating *Cynanchum auriculatum* Royle ex Wight water extract (**A**: Liver from male control group, **B**: Liver from 2000 mg/kg/day treatment group (periportal microvesicular vacuolation hepatocyte))

Compared to control group, male rat group exposed to 2000 mg/kg/day of *C. auriculatum* Royle ex Wight water extracts (CAE) showed increased triglyceride level (86 ± 16 mg/dL, *p* < 0.05) at 59.2% and absolute (16.63 ± 1.59 g, p < 0.01) and relative (2.74 ± 0.14 g/100 g bw, *p* < 0.01) liver weight at 14.5, 8.7% respectively. Additionally, periportal microvesicular vacuolation, hepatocyte was observed in histopathological findings, and its occurrence frequency and severity in hepatocyte were also elevated (Table 2, Fig. 3). Administering more than 1000 mg/kg/day of *C. auriculatum* Royle ex Wight water extract (CAE) precipitated elevated triglyceride and absolute/relative liver weights in male rat group indicating liver damage. Along with increased triglyceride levels and liver weights, it could be concluded that these findings indicated liver dysfunction. In conclusion, NOAEL was 1000 mg/kg/day and the target organ was liver in male rats while NOAEL for female rat was above 2000 mg/kg/day and target organ was not selected.

**Table 2:** Significant changes and histopathology results for male rats in 90-day gavage study of *Cynanchum auriculatum* Royle ex Wight water extract

	Control	500 mg/kg/day	1000 mg/kg/day	2000 mg/kg/day
Clinical chemistry				
Triglyceride (mg/dL)	54 ± 27	79 ± 34	68 ± 17	86 ± 16*
Organ weights				
Liver				
Absolute (g)	14.52 ± 1.46	16.57 ± 1.39**	15.64 ± 1.29	16.63 ± 1.59**
Relative (g/100 g bw)	2.52 ± 0.14	2.64 ± 0.12	2.60 ± 0.16	2.74 ± 0.14**
Histopathology				
Liver				
Periportal microvesicular vacuolation, hepatocyte	10	7	8	10
Minimal	7	6	3	1
Slight	1	–	5	7
Moderate	2	1	–	2

*,**Significantly different from control group by Dunnett’s t-test (*p* < 0.05, *p* < 0.01)

**Fig. 4:**
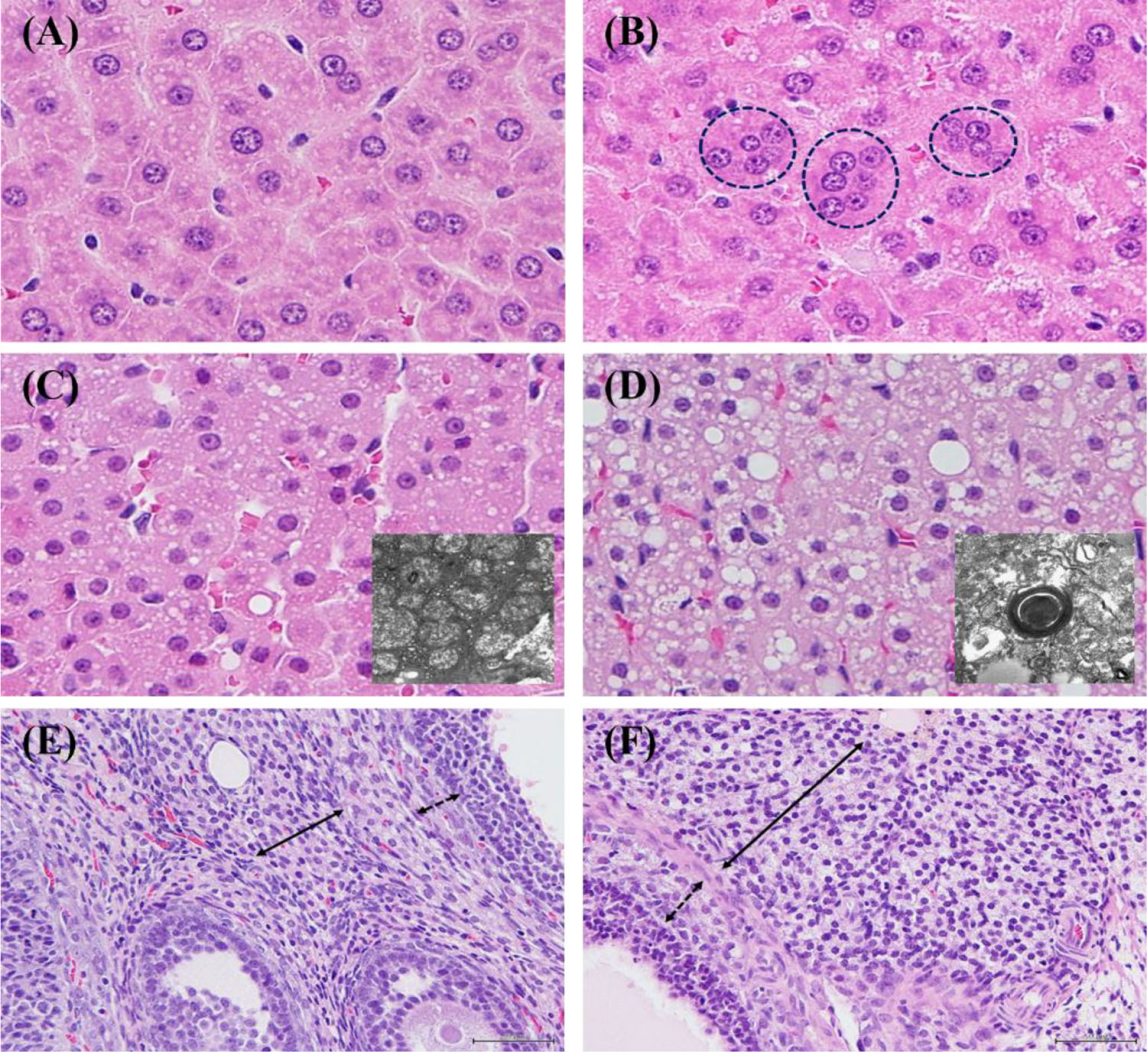
Histopathological examinations treating *Cynanchum auriculatum* Royle ex Wight powder (**A**: Liver from male control Group, **B**: Liver from 500 mg/kg/day treatment group (Multinucleated hepatocytes), **C**: Adrenal gland from female control group, **D**: Adrenal gland from 500 mg/kg/day treatment group (Hypertrophy/vacuolation, zona fasciculata), **E**: Ovary from female control group, **F**: Ovary from 500 mg/kg/day treatment group (Vacuolation, interstitial and theca cell))

After treating *C. auriculatum* Royle ex wight powder, increased alanine aminotransferase (ALT) (207.5 ± 144.8, *p* < 0.01), aspartate aminotransferase (AST) (484.2 ± 417.2, *p* < 0.01) levels in serum were recognized in 500 mg/kg/day and above male group. Along with these signs, notable histopathological findings indicating liver damage like single cell necrosis or multinucleated hepatocytes were also observed. Meanwhile, in 500 mg/kg/day female group, absolute and relative weights of adrenal glands went heavier and hypertrophy/vacuolation, zona fasciculata were detected in histopathological findings (Tables 3, 4). Moreover, vacuolation, interstitial and theca cells in ovaries were also detected (Fig. 4). As a result, NOAEL was 150 mg/kg/day in both male and female group and the target organ was liver in male and adrenal glands and ovaries in female.

**Table 3:** Significant changes for rats in 90-day gavage study of *Cynanchum auriculatum* Royle ex Wight powder

	Control	50 mg/kg/day	150 mg/kg/day	500 mg/kg/day
n	10	10	10	10
Male				
Clinical chemistry				
Alanine aminotransferase (U/L)	30.3 ± 5.9	29.6 ± 4.3	33.5 ± 11.0	207.5 ± 144.8**
Aspartate aminotransferase (U/L)	95.2 ± 22.2	96.3 ± 21.5	93.4 ± 16.8	484.2 ± 417.2**
Female				
Organ weights				
Adrenal glands				
Absolute (g)	0.0702 ± 0.0077	0.0751 ± 0.0082	0.0719 ± 0.0091	0.0883 ± 0.0105**
Relative (g/100 g bw)	0.0223 ± 0.0025	0.0249 ± 0.0025	0.0237 ± 0.0020	0.0301 ± 0.0035**

**Significantly different from control group by Dunnett’s t-test (*p* < 0.01)

**Table 4:** Histopathological findings for rats in 90-day gavage study of *Cynanchum auriculatum* Royle ex Wight powder

	Control	50 mg/kg/day	150 mg/kg/day	500 mg/kg/day
n	10	10	10	10
Male				
Liver				
Multinucleated hepatocyte	0	0	0	2
Single cell necrosis	0	0	0	5***
Female				
Adrenal glands				
Hypertrophy/vacuolation, zona fasciculata	0	0	0	3***
Ovaries				
Vacuolation, interstitial and theca cells	0	0	0	5***

^***^Significantly different from control group by Kruskal–Wallis and Mann–Whitney U-test (*p* < 0.05)

In contrast to our results, Ding et al. (2019) reported pretreatment of CA extracted fraction, mainly composed of C-21 steroidal glycosides, could attenuate liver injuries from diethylnitrosamine, along with decreased serum biochemical indicators like AST, ALT and etc. Wu et al. (2018) also reported C-21 steroidal glycosides extracted from the roots of CA showed liver protective effects from hydrogen peroxide by radical-scavenging. However, in these two tests, CA was extracted not only by ethanol, but also by chloroform and ethyl acetate. Vacuolation in adrenal glands and ovaries, observed in groups treated with CA powder, indicated phospholipidosis (Dixon et al., 2014; Gopinath et al., 2012; Sahota et al., 2013). Even though there are several studies of CW about lipid metabolism, to our knowledge, related CA studies are not published yet.

Following the food safety incident in May 2015, Ministry of Food and Drug Safety then immediately initiated toxicity tests. Following the results of toxicity studies, in December 2017, CW was consequently restricted to water extract only, and CA remains prohibited in Korea. Several researches and studies about beneficial aspects of CW and CA, and its components have been reported since then. Especially, liver protection (Qin et al., 2021) and lipid profile control (Shin et al., 2020) of CH and CA were published in recently conducted in vitro tests and clinical trials. Still, toxicities of CW and CA were not been clearly identified. Even though further research will be required to identify the mode of action and components which cause the adverse effect, to the best of our knowledge, this is the first sub-chronic toxicity study following GLP protocols on the adverse effects, target organs and its dose levels of *C. wilfordii* (Maxim.) Hemsley and *C. auriculatum* Royle ex Wight.
